# Should We Stop Collecting the Preoperative Autologous Blood before Bone Marrow Harvest?

**DOI:** 10.3390/jcm10102134

**Published:** 2021-05-14

**Authors:** Daniel Lysák, Lenka Hejretová, Marcela Hrabětová, Pavel Jindra

**Affiliations:** 1 Department of Hematology and oncology, University Hospital Pilsen, Edvarda Beneše 1128/13, 305 99 Pilsen, Czech Republic; hejretoval@fnplzen.cz (L.H.); hrabetovam@fnplzen.cz (M.H.); jindra@fnplzen.cz (P.J.); 2 Faculty of Medicine in Pilsen, Charles University, Husova 3, 301 00 Pilsen, Czech Republic; 3 Czech National Marrow Donors Registry, Na Roudné 123/212, 301 00 Pilsen, Czech Republic

**Keywords:** bone marrow, preoperative autologous blood, harvest, allogeneic donor, CD34+ cells

## Abstract

Preoperative autologous blood donation (PAD) in bone marrow (BM) donors is performed to meet potential post-harvest transfusion needs and to avoid the risk of allogeneic transfusions. We reviewed retrospectively bone marrow harvests in 216 healthy donors during a ten-year period to determine the use of autologous blood. All donors except four had undergone PAD. The initial hemoglobin level of 153 g/L (male donors) and 135 g/L (female donors), respectively, decreased by about 8 g/L after preoperative blood donation and by 23 g/L after bone marrow harvest (medians). Autologous blood was administered to 70% of donors, 30% of the units remained unused. The evaluation of the risk of reaching transfusion threshold (<115 g/L males, <105 g/L females) revealed that donors with initial hemoglobin above 145 g/L and those weighing above 75 kg have minimal risk of requiring blood substitution (about 10%). A larger volume of bone marrow was obtained from male compared to female donors (1300 vs. 1100 mL) because of their higher body weight, which resulted in a higher number of procured nucleated cells (362 vs. 307 × 10^6^/kg TNC, ns). The donor-recipient weight difference predicted the probability of sufficient collection. Only 1.5% of donors weighing ≥ 20 kg more than recipients failed to reach ≥3 × 10^8^/kg TNC recipient. Our findings affirm previous data that PAD is unnecessary for healthy marrow donors and may be indicated individually after considering the pre-collection hemoglobin level, donor and recipient weight, and expected blood loss. Reasonable substitution cut-offs have to be set together with clinical symptom evaluation. The effective use of PAD also requires an adequate time interval between PAD and BM harvest.

## 1. Introduction

Bone marrow (BM) harvest from related or unrelated donors is a standard procedure used through years of hematopoietic stem cell transplant program. The goal of the procedure is to obtain enough hematopoietic stem cells with minimal potential risks to the volunteer donors. The major side effects are pain at the collection site (82% of donors) and pains associated with anesthesia (sore throat, headache) followed by weakness and fatigue [1].

The substantial withdrawal of red blood cells along with bone marrow collection causes a decrease in hemoglobin and induces the risk of anemic symptoms [2]. Preoperative autologous blood donations (PAD) are performed to supplement post-harvest hemoglobin decline, improve the post-donation recovery, and avoid the risk associated with allogeneic blood. The practice of PAD was introduced during the 1980s and 1990s due to increased awareness of transfusion-transmitted infectious diseases (HIV, hepatitis). The increasing safety of allogeneic transfusions changes the perception of the risk-benefit ratio of this practice. Several published studies question the routine PAD use during BM harvest [3,4,5]. The policy for autologous blood collection varies between transplant centers. Because of its low clinical usability, some centers do not collect autologous blood [6], while others perceive PAD as a routine procedure [7].

The autologous blood collection contributes to anemia before BM harvest, causes over-transfusion, which may add unnecessary transfusion-related risks to donors, and increases blood wastage, as at least one-third of PAD is not administered [3,4,5]. Poor timing of blood collection may reduce preoperative hemoglobin levels. A study in healthy donors showed that recovery of hemoglobin to 80% of baseline after withdrawing 500 mL of blood takes approximately 30 days with iron substitution [8].

Nowadays, the World Marrow Donor Association (WMDA) does not specify PAD use within the standards (WMDA standards 2020). JACIE standards require the availability of autologous or irradiated allogeneic blood (FACT-JACIE standards edition 7). Policies applied by many transplant centers reflect this trend, and autologous blood is not collected at all, or the collection decisions are guided by expected blood loss and the required number of stem cells [9].

We evaluated bone marrow harvests performed over a ten-year period. The aim was to analyze the kinetics of hemoglobin after autologous blood and bone marrow collection and to identify donors who may benefit from preoperative blood collection and those with a low probability of PAD need. The study should clarify the indication for PAD in marrow donors and help to update our substitution protocol.

## 2. Donors and Methods

In a retrospective study, we analyzed bone marrow harvests performed from 2008 to 2018 in 216 healthy, related and unrelated donors (160 men and 56 women). Two to four weeks before collection, all donors underwent a pre-collection examination (work-up). At the same time, one unit of PAD (350–450 mL) was taken from all donors. Only in 4 donors, no autologous erythrocytes were prepared (3 collections for a pediatric recipient, 1 unplanned collection in case of failure of peripheral stem cell mobilization). The median age of donors was 32 years (19–63), weight 82 kg (50–131). Donor characteristics are shown in Table 1.

Bone marrow collection was performed in an aseptic operating room environment under general anesthesia. The technique comprised repeated aspirations with trephine biopsy needles from the posterior iliac crest. The maximum collected amount did not exceed 20 mL/kg of donor weight or 1350 mL of bone marrow. The collection methodology complied with the applicable national regulation, the standards of the Czech National Marrow Donor Registry, and the WMDA. The administration of autologous blood during the procedure was decided on the basis of the initial hemoglobin level, blood loss, and vital functions. When blood was not administered in the operating room, the decision for substitution was made 8–10 h after BM collection based on a repeated blood count.

Informed consent was obtained from all donors in accordance with the suitable clinical practice. The donors have agreed to the use of data obtained during the donation process for procedure validations as well as for scientific purposes. The study was approved by the medical director and quality management board.

### Statistics

Basic statistical parameters were calculated for the measured parameters. Spearman’s correlation coefficient and linear regression were used to determine the dependencies of the investigated parameters. Factors that showed statistical significance with respect to the monitored endpoints were detected using multidimensional regression and then processed into classification and regression trees (CART) using logistic regression. Statistical significance was determined at alpha = 5%. Statistical analysis was performed using SAS software (SAS Institute Inc., Cary, NC, USA).

## 3. Results

The total collection time ranged from 15 to 95 min (median 35 min). The median amount of bone marrow collected was 1170 mL (445–1350 mL) without counting anticoagulants, corresponding to 15 mL/kg donor weight (4–20 mL/kg). Higher volume, approximately 200 mL, was collected from males relative to females (median 1295 vs. 1100 mL, *p* = 0.015), which is due to differences in donor weight (86 vs. 65 kg, *p* < 0 0001). The total amount of nucleated cells (TNCs) obtained per kilogram of the recipient was slightly higher in male donors but without reaching statistical significance (Table 1).

The hemoglobin (Hb) level at work-up was 153 g/L in male and 135 g/L in female donors (medians, *p* < 0.0001). It decreased approximately by 8 g/L after PAD and by another 23 g/L (male) or 25 g/L (female) after BM harvest. Only 13% of donors experienced a decrease in hemoglobin to 90–100 g/L, and only one donor (0.6%) dropped below 90 g/L. Autologous blood was administered to 70% of donors, 25% of donors were substituted already in the operating room, and 75% of them in the evening after blood count examination. A total of 30% of the blood units remained unused. Hemoglobin kinetics data are shown in Table 2.

**Figure 1:**
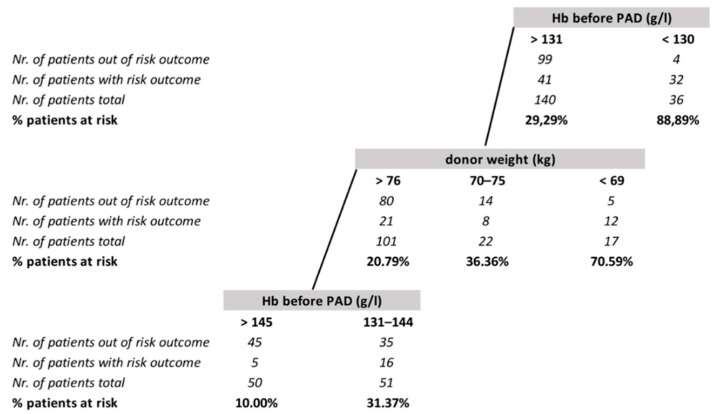
CART model for the risk of Hb decrease below 115 g/L (male) resp. 105 g/L (female) after bone marrow harvest.

We tried to characterize the group of donors who were at risk of a hemoglobin decrease below 115 g/L (male) or 105 g/L (female), which corresponded to our substitution trigger. The probability of transfusion was higher in donors with lower pre-collection hemoglobin levels. Almost 90% of donors with pre-collection Hb ≤ 131 g/L received autotransfusion, while for donors with Hb above 131 g/L, the donor’s weight was identified to be another refinement factor. The probability of substitution ranged from about 20% for donors over 76 kg to 70% for donors below 69 kg. The need for autotransfusion decreased to 10% for donors that both weighed more than 76 kg and had Hb above 145 g/L (Figure 1). All these evaluations were performed only for donors who did not receive autologous blood in the operating room (176 donors).

**Figure 2:**
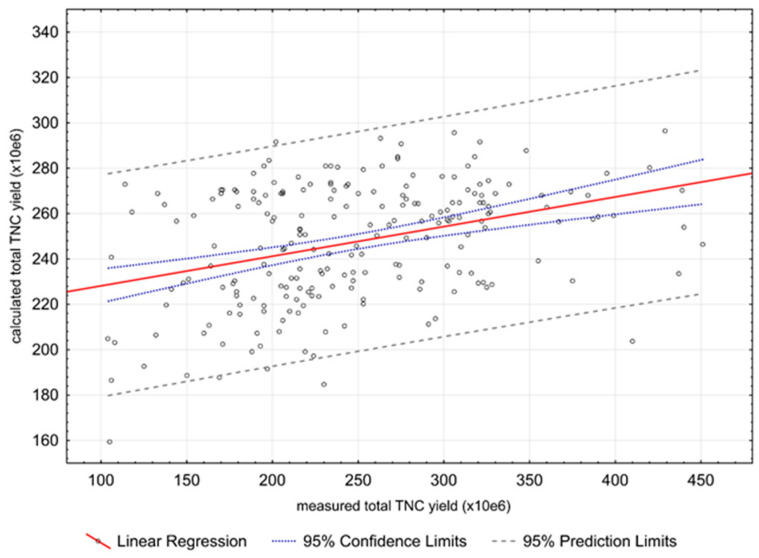
Comparison of calculated and measured collection yields (TNC ×10^6^), linear regression.

We also analyzed parameters that affected the yield of the total nucleated cells. Various models were tested using multidimensional regression, and two independent variables were found that best predicted the harvest yield: marrow harvested volume (*p* < 0.0001) and donor weight (*p* = 0.01). The amount of TNC improved with increasing donor weight and with the volume of marrow collected. Predicted yields calculated according to the model (TNC = 75.88 + 0.09 * volume BM + 0.81 * donor weight) correlated well with the measured values (*p* < 0.0001; r = 0.34) (Figure 2).

**Figure 3:**
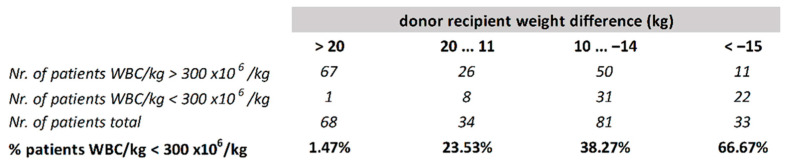
CART model for the probability of bone marrow harvest with TNC content ≤ 3 × 10^8^/kg recipient depending on the weight difference between the donor and recipient.

The median amount of TNC required by the transplantation centers was 3 × 10^8^/kg recipient weight. The only statistically significant factor predicting the failure to reach this yield was the weight difference between the donor and the recipient. Donors weighing 20 kg or more than the recipients had only a 1.5% chance of the collection being below 3 × 10^8^/kg. The risk increased gradually with decreasing donor weight up to 67% for donors weighing ≥ 15 kg less than the recipients (Figure 3).

## 4. Discussion

Historically, PAD circumvented possible administration of allogeneic erythrocytes and thus lowered the risk of blood-borne infections in healthy donors. The improvement in donor testing and increased safety of allogeneic blood supply has led to a decline in the use of autologous blood. Preoperative autologous blood donation in BM donors continues to be disputed; however, widely accepted guidelines are yet to be defined. There is an ongoing discussion on the rationale, safety, and cost-effectiveness of routine PAD before BM harvest.

Surveys conducted by U.S. transplant centers revealed that about 23 to 50% of centers do not routinely collect any autologous blood [1,9], while other centers, including ours, prepare PAD for most donors [4,10,11]. The number of autotransfusions that are not administered and disposed of can reach 25% or even 50% in some cases [3,5,7,12]. In our series, we used autologous blood for 70% of donors, i.e., 30% of the units were wasted.

Autologous blood use is not completely without risk. PAD collection reduces preoperative hemoglobin level by up to 20 g/L (median in our cohort 8 g/L) depending on the number of units and time to BM harvest [3,4,11]. PAD can expose marrow donors to a risk of preoperative anemia and hence a greater risk of transfusion. The eventual benefit of PAD can be achieved only if an adequate interval between blood and marrow collection is permitted, allowing sufficient time for erythrocyte regeneration.

Frequent whole blood donors are prone to iron deficiency from progressive blood loss. The strategy to mitigate donation-induced iron loss includes oral iron supplementation [13]. Iron supplementation seems to be reasonable in bone marrow donors, especially in those with a lower initial hemoglobin level. The administration of iron during the period before and after marrow collection can provide the space for red blood cell recovery. Erythropoiesis stimulating agents were developed to help reduce the need for allogeneic blood transfusion in patients with end-stage chronic kidney disease, chemotherapy-induced anemia, or undergoing elective surgery [14]. However, the use of erythropoietin in healthy stem cell donors to maintain hematocrit levels is generally not recommended. The need for PAD or the risk of allogeneic blood administration is also determined by the hemoglobin level that is established as the threshold for substitution. If we accept the 70–80 g/L hemoglobin as a substitutional cut-off value recommended for patients without cardiovascular or respiratory diseases [15], the number of donors requiring transfusion will be significantly reduced. Bartnik et al. showed that only 1,3% of donors dropped below 80 g/L after BM harvest, and none of them below 70 g/L [16]. Similar conclusions were made by Parkkali et al., who reported post-harvest hemoglobin below 85 g/L only in 2% of donors [7]. According to our experience, the hemoglobin levels decreased to 90–100 g/L in 13% of donors, and only one donor (0.6%) had a post-harvest hemoglobin level below 90 g/L. Female donors (91%) and donors weighing less than the recipient (61%) were overrepresented in this group. While the 80 g/L hemoglobin level is certainly sufficient for clinically stable patients, we should apply a more liberal transfusion policy for healthy donors. Low hemoglobin levels may lead to symptoms of weakness, discomfort, nausea, vomiting, or prolonged hospitalization. Our goal is not only to protect the health of the donors but also to maintain their comfort and quality of life. Although there are no clear long-term differences in the health-related quality of life experiences of the bone marrow and peripheral blood stem cells (PBSC) donors, bone marrow donation is associated with more short-term negative quality of life effects [17,18]. BM donors experience more fatigue and less energy in the first week after donation than PBSC donors [18,19]. Therefore, for a specific subgroup of donors with unfavorable characteristics (lower initial hemoglobin and/or significant weight difference), transfusion is still appropriate.

Transplant centers apply different substitution policies. Many centers routinely transfuse autologous blood regardless of post-operative hemoglobin level [4,9,10], while other reinfuse the autologous blood depending on the preoperative Hb level, the volume of BM collected, and donors’ vital signs [3]. Our practice is to administer autotransfusion in the operating room only in case of clinically significant blood loss or when the preoperative hemoglobin is low (<125 g/L). Otherwise, substitution is considered based on a blood count examined 8–10 h after BM harvest. Autotransfusion is provided when hemoglobin drops below 105 g/L in female and below 115 g/L in male donors, when hemoglobin decreases by 30–35 g/L from the pre-collection value, or in the case of poor tolerance of blood loss. A similar substitution Hb level (109 g/L) was reported by Farhadfar et al. [5].

The evaluation of factors that predict a decrease in Hb after collection below these values can identify donors who have a low probability of red blood cell transfusion. Our experience shows that donation of PAD is unnecessary for donors with a sufficiently high level of hemoglobin (~145 g/L) and a sufficient weight (~76 kg) or a significant weight disproportion in favor of the donor (~20 kg). If we use even a lower cut-off, the percentage of donors able to undergo bone marrow collection without autotransfusion will grow. The hemoglobin cut-off <145 g/L as a predictor of the need for transfusion after BM harvest was also identified by Gilli et al. [11].

To date, there is a consensus that routine PAD prior to bone marrow collection is not required. Transplant centers should have a defined policy that specifies a population of donors for whom PAD is recommended. These rules should be based on donor and recipient parameters, the expected volume of bone marrow collected, and post-harvest monitoring.

## 5. Conclusions

Autologous blood collection is no longer routinely recommended for bone marrow collection, and its use is progressively declined over the last decade. Donors potentially benefiting from PAD can be prospectively identified based on their preoperative Hb level, expected volume of bone marrow harvest, and donor-recipient weight ratio. A sufficient interval (at least 3 weeks) must be set between PAD and BM harvest to enable the recovery of Hb drop subsequent to PAD, together with iron supplementation during the peri-collection period. Availability of autologous blood or a decrease in hemoglobin should not trigger a transfusion. Rather the donors’ clinical symptoms should always be taken into account. On the other hand, maintaining a sufficient hemoglobin level in the donor, who is at risk of its higher decline, may minimize the reduction in the donor’s quality of life in the post-collection period.

Nonetheless, neither a restrictive nor a liberal transfusion policy can be selected unilaterally due to the absence of long-term data measuring the impact of different substitution approaches on donors’ quality of life and day-to-day performance or morbidity.

## Figures and Tables

**Table 1:** Donors and collections characteristics.

	All Donors	Male	Female	*p*
Number of donors	216	160	56	na
Males/females	100%	74%	26%	na
Age (years)	32 (19–63)	32 (19–63)	29 (19–47)	ns
Weight (kg)	82 (50–131)	86 (63–131)	65 (50–100)	*p* < 0.0001
Bone marrow harvest volume (mL)	1170 (445–1350)	1295 (445–1350)	1100 (450–1350)	*p* = 0.015
Marrow volume/kg donor (mL)	15 (4–20)	14 (4–20)	17 (8–20)	*p* < 0.0001
Collected TNC/kg patient (×10^6^/kg)	347 (124–1971)	362 (156–1625)	307 (124–1971)	ns

Values are given as medians; abbreviations: TNC—total nucleated cells.

**Table 2:** Changes in hemoglobin levels during autologous blood and bone marrow collections.

	All Donors	Male	Female	*p*
Hb at work-up (g/L)	149 (103–175)	153 (103–175)	135 (112–152)	*p* < 0.0001
Hb day before BM harvest (g/L)	141 (100–168)	153 (127–175)	126 (100–139)	*p* < 0.0001
Hb decrease after PAD collection (g/L)	8 (0–34)	8 (0–34)	9 (0–30)	ns
Hb after BM (AT yes *) (g/L); 111 donors	111 (86–145)	116 (95–145)	100 (86–118)	*p* < 0.0001
Hb after BM (AT no) (g/L); 67 donors	124 (103–146)	125 (111–146)	106 (103–113)	*p* = 0.0001
Hb decrease after BM collection (g/L)	23 (4–56)	23 (4–56)	25 (4–50)	ns

Values are given as medians; abbreviations: AT—autotransfusion, BM—bone marrow. * AT 8–10 h after BM harvest.

## Data Availability

The data that support the findings of this study are available upon request from the corresponding author.

## References

[1] Miller J.P., Perry E.H., Price T.H., Bolan C.D., Karanes C., Boyd T.M., Chitphakdithai P., King R.J. (2008). Recovery and safety profiles of marrow and PBSC donors: Experience of the national marrow donor program. Biol. Blood Marrow Transpl..

[2] Manuel S.P., Spitzer T.R., Ishikawa Y. (2017). Preoperative autologous blood donation in healthy bone marrow donors contributes to pre-procedure anemia. Bone Marrow Transpl..

[3] Arora K., Kelley J., Martinez F., Tholpady A. (2018). Preoperative autologous blood collection before bone marrow harvest in haploidentical related donors: Is it justified?. Transfusion.

[4] Teofili L., Valentini C., Bianchi M., Pellegrino C., Bellesi S., Chiusolo P., Laurenti L., Innocenti I., De Stefano V., Bacigalupo A. (2019). Preoperative autologous blood donation in adult bone marrow donors: Reappraisal of a single- centre experience. Vox Sang..

[5] Farhadfar N., Murthy H., Logan B., Sees J., Mouhab A., Battiwala M., Beitinjaneh A.M., Chhabra S., Diaz M.A., Engels K. (2020). Impact of autologous blood transfusion after bone marrow harvest on unrelated donor´s health and outcome: A CIBMTR analysis. Bone Marrow Transpl..

[6] Pruszczyk K., Skwierawska K., Król M., Moskowicz A., Jabłoński D., Torosian T., Piotrowska I., Urbanowska E., Wiktor-Jędrzejczak W., Snarski E. (2017). Bone marrow harvest from unrelated donors-up-to-date methodology. Eur. J. Hematol..

[7] Parkkali T., Juvonen E., Volin L., Partanen J., Ruutu T. (2005). Collection of autologous blood for bone marrow donation: How useful is it?. Bone Marrow Transpl..

[8] Kiss J.E., Brambilla D., Glynn S.A., Mast A.E., Spencer B.R., Stone M., Kleinman S.H., Cable R.G. (2015). National heart, lung, and blood institute (NHLBI) recipient epidemiology and donor evaluation study-III (REDS-III). Oral iron supplementation after blood donation: A randomized clinical trial. JAMA.

[9] Spitzer T.R., Sugrue M.W., Gonzalez C., O’Donell P., Confer D., Pulsipher M.A., Schwartz J., Linenberger M. (2017). Transfusion practices for bone marrow harvests: A survey analysis from the AABB Bone Marrow Quality Improvement Initiative Working Group. Bone Marrow Transpl..

[10] Fujiwara S., Ikeda K., Kino S., Tanaka A., Hasegawa Y., Fujino K., Makino S., Matsumoto M., Yokohama A., Takeshita A. (2020). Clinical significance of autologous blood transfusion in bone marrow harvest from unrelated donors. Int. J. Hematol..

[11] Gilli I., Vigorito A., Benites B. (2019). Revisiting old practices: More restricted indication of preoperative autologous blood donation in healthy bone marrow donors according to baseline hemoglobin levels. Trans. Apher. Sci..

[12] Gouëzec H., Ferré N., Hervé F., Lapart C., Bernard M., Dauriac C., Nimubona S. (2015). Suitability of autologous blood donation before bone marrow donation. Transfus. Clin. Biol..

[13] Kiss J.E., Vassallo R.R. (2018). How do we manage iron deficiency after blood donation?. Br. J. Hematol..

[14] Goodnough L.T., Shander A. (2013). Current status of pharmacologic therapies in patient blood management. Anesth. Analg..

[15] Carson J.L., Grossman B.J., Kleinman S., Tinmouth A.T., Marques M.B., Fung M.K., Holcomb J.B., Illoh O., Kaplan L.J., Katz L.M. (2012). Red blood cell transfusion: A clinical practice guideline from the AABB. Ann. Intern. Med..

[16] Bartnik K., Pruszczyk K., Skwierawska K., Król M., Płachta M., Moskowicz A., Zakrzewski T., Urbanowska E., Jędrzejczak W.W., Snarski E. (2018). Bone marrow harvest in donors with anaemia. Vox Sang..

[17] Fujimoto A., Suzuki R., Orihara K., Lida M., Yamashita T., Nagafuji K., Kanamori H., Kodera Y., Miyamura K., Okamoto S. (2020). Health-related quality of life in peripheral blood stem cell donors and bone marrow donors: A prospective study in Japan. Int. J. Hematol..

[18] Switzer G.E., Bruce J.G., Harrington D., Haagenson M., Drexler R., Foley A., Confer D., Bishop M., Anderlini P., Rowley S. (2014). Health-related quality of life of bone marrow versus peripheral blood stem cell donors: A prespecified subgroup analysis from a phase III RCT—BMTCTN protocol 0201. Transplant. Cell. Ther..

[19] Bredeson C., Leger C., Couban S., Simpson D., Huebsch L., Walker I., Shore T., Howson-Jan K., Panzarella T., Messner H. (2004). An evaluation of the donor experience in the canadian multicenter randomized trial of bone marrow versus peripheral blood allografting. Transplant. Cell. Ther..

